# Long-term follow-up of children with neuroblastoma receiving radiotherapy to metastatic lesions within the German Neuroblastoma Trials NB97 and NB 2004

**DOI:** 10.1007/s00066-020-01718-5

**Published:** 2020-12-09

**Authors:** Danny Jazmati, Sarina Butzer, Barbara Hero, Jerome Doyen, Dalia Ahmad Khalil, Theresa Steinmeier, Stefanie Schulze Schleithoff, Angelika Eggert, Thorsten Simon, Beate Timmermann

**Affiliations:** 1 Department of Particle Therapy, West German Proton Therapy Centre Essen (WPE), West German Cancer Center (WTZ), University Hospital Essen, Hufelandstraße 55, 45147 Essen, Germany; 2 Children’s Hospital, University of Cologne, Cologne, Germany; 3 Department of Pediatrics, Division of Oncology and Hematology, Charité—Universitätsmedizin Berlin, corporate member of Freie Universität Berlin, Humboldt-Universität zu Berlin, and Berlin Institute of Health, Charitéplatz 1, 10117 Berlin, Germany; 4 Department of Particle Therapy, West German Proton Therapy Centre Essen (WPE), West German Cancer Center (WTZ), German Cancer Consortium (DKTK), University Hospital Essen, Essen, Germany

**Keywords:** Childhood cancer, Survival, Clinical trial, Radiation oncology, Stage 4

## Abstract

**Purpose:**

Neuroblastoma (NB) is the most common extracranial solid malignancy during childhood. Despite a multimodal treatment approach, the prognosis of patients with metastatic NB is not satisfactory. Although radiotherapy (RT) has become an integral part of treatment of the primary tumor, the role of RT in osteomedullary lesions is not well defined. A retrospective analysis was conducted to evaluate the impact of RT for metastatic sites in children with high-risk NB.

**Methods:**

All patients with stage 4 NB from the prospective, multicenter NB trials NB97 and NB2004 who received RT to metastatic sites during frontline treatment were included in this retrospective analysis.

**Results:**

A total of 18 children were irradiated with a median dose of 36 Gray (Gy; range 20–45 Gy) to one or more (range 1–3) osteomedullary metastases with or without concomitant RT to the primary tumor site. The median follow-up time was 149 months (range 55–220) in survivors. At 5 years, local relapse-free survival (LRFS) at irradiated metastatic sites and metastases-free survival (MFS) at distant, non-irradiated site rates were 51.4 and 39.9%, respectively. The estimated overall survival (OS) rate at 5 years was 49.4%. No high-grade acute or late toxicity and no secondary malignancy was reported.

**Conclusion:**

RT to metastases is feasible for patients with stage 4 NB. However, an impact of RT to residual metastatic sites on outcome was not found. Studies with larger cohorts or prospective trials would be desirable in order to elucidate the role of RT for metastases.

## Introduction

Deriving from the developing sympathetic nervous system, neuroblastoma (NB) is the most common extracranial solid tumor in children, accounting for 7–10% of all pediatric malignancies [1]. Patients are classified and treated according to different risk groups based on age, stage, and molecular pathology. Despite a multimodal risk stratification and multimodal treatment approaches comprising different combinations of chemotherapy, surgery, stem cell transplantation (SCT), ^131^I‑meta-iodobenzylguanidine (MIBG) therapy, radiotherapy (RT), isotretinoin or anti-GD2-directed antibodies, the prognosis of high-risk patients remains poor with a 5-year overall survival (OS) below 50% [2–4]. More than 50% of all NB patients present with metastatic lesions at diagnosis, predominantly osteomedullary (bone marrow, 70.5%; bone, 55.7%), in distant lymph nodes (30.9%), and in the liver (29.6%) [5]. Disseminated disease in patients older than 18 months is usually associated with an unfavorable prognosis despite intensive therapy [6, 7]. Given the high radiosensitivity of NB, irradiation has become an integral part for treatment of the primary tumor in children with high-risk NB. It is discussed whether applying focal RT to metastatic sites of disease could improve outcome. However, this has never been addressed in a randomized trial, although some series have demonstrated improved control rates [8–11]. So far, current treatment strategies in Germany do not recommend regular irradiation of metastatic sites, but mostly allow individual decisions in case of limited residual osteomedullary metastases. In contrast, the American COG trial “A Phase III Randomized Trial of Single versus Tandem Myeloablative Consolidation Therapy for High-Risk Neuroblastoma” (ANBL0532) proposes to irradiate up to five persisting metastatic sites with 21.6 Gray (Gy) [12].

The aim of the present study was to analyze the outcome of patients irradiated to residual osteomedullary metastatic sites during frontline treatment within the trials “Neuroblastom NB 97” (NB97) (EU – 20661) and “Neuroblastom 2004 Trial Protocol for Risk Adapted Treatment of Children with Neuroblastoma” (NB2004) (NCT 00410631; NCT 00526318) of the German Society for Pediatric Oncology and Hematology (GPOH).

## Methods

### Patients

All patients with metastatic osteomedullary lesions irradiated during frontline therapy treated within the prospective, multicenter NB trials “Neuroblastomstudie NB 97” (NB97; EU-20661) or “NB2004 Trial Protocol for Risk Adapted Treatment of Children with Neuroblastoma” (NB2004; NCT 00410631; NCT 00526318) were included in this analysis. The retrospective data assessment was conducted between 11.2018 and 05.2020. In compliance with the Helsinki declaration and its later amendments, all parents of participating patients had consented to participation in the respective trials. The Institutional Ethical Board of the University of Cologne had approved the trial protocols.

### Treatment

Induction chemotherapy consisted of three N5 (cisplatin, etoposide, and vindesine) and three N6 cycles (vincristine, dacarbacine, ifosfamide, and doxorubicine), as reported elsewhere, for patients treated within NB97 and for the standard arm of NB2004 [13]. In the experimental high-risk arm of the NB2004 trial (NB2004-HR), two additional TCE cycles (topotecan, cyclophosphamide, and etoposide) were given prior to the standard induction chemotherapy [14].

After induction chemotherapy, either maintenance treatment (NB97, NB2004 intermediate risk) or high-dose myoablative chemotherapy followed by autologous stem cell rescue (NB97, NB2004-HR) was scheduled. Post-consolidation treatment consisted of immunotherapy with the antibody ch14.18 (NB97) or retinoic acid (NB97, NB2004), as reported elsewhere [2]. Surgery of the primary lesion was scheduled during induction treatment. In case of residual MIBG uptake at the primary or metastatic site at the end of induction chemotherapy, MIBG therapy was scheduled prior to high-dose treatment [15].

In accordance with the NB97 and NB2004 protocols, external beam radiotherapy (EBRT) to a total dose of 36–40 Gy was applied with a daily fractional dose of 1.6–2.0 Gy to the active residual primary, defined either as MIBG uptake or, in case of MIBG-negative, as contrast-enhancement of the primary residual [16]. RT of osteomedullary metastases was not part of the protocol but was performed based on individual decision.

### Toxicity

Acute and late high-grade toxicities were reported to the study center if they were attributable to RT.

### Statistical analysis

Qualitative data were presented as frequency (minimum–maximum) and percentage. Cut-off was based on known cut-off or median. Local relapse (LR) was used to describe the failure rates at irradiated metastatic lesions. Accordingly, local relapse-free survival from diagnosis (LRFS) was defined as the absence of local relapse. Metastatic failure (MF) was defined as a metastatic relapse occurring at a non-irradiated metastatic site. Local relapse-free survival (LRFS), metastasis-free survival (MFS), and OS were calculated and plotted according to the Kaplan–Meier method. Patients were censored at the time of last follow-up if they had no event. All statistical analyses were performed using Statistical Package for the Social Sciences (SPSS, SPSS Inc., Chicago, IL, USA) version 16.0 on Windows®. Log-rank analysis was performed in order to identify potential factors correlated with control rates.

## Results

### Patient cohort and treatment

Eighteen patients with stage 4 NB having received irradiation to a total of 24 metastatic osteomedullary lesions were included in this analysis. The median age of the patients at diagnoses was 46 months (range 11–175). Ten patients had been treated in the high-risk arm of the NB97 trial, seven in the high-risk arm of the NB2004 trial, and one infant with stage 4 NB in the intermediate-risk arm of the NB2004 trial. Details on patient and disease characteristics are displayed in Table 1. Seventeen patients presenting with 53 lesions detected before starting RT received irradiation to 23 MIBG-active metastatic lesions, whereas one patient received RT to one metastasis after complete remission due to previous therapy. The irradiated metastatic sites were located either in the skull (*n* = 7), extremity (*n* = 10), spine (*n* = 5), or ribs (*n* = 2). Details on treatment characteristics are described in Table 2. RT to metastatic lesions was scheduled according to the time of RT to the primary tumor. Radiation to metastatic lesions was applied either alone or concurrently with RT to the primary site in patients with MIBG-active residual primary tumors (*n* = 2). The decision to irradiate metastases was taken individually by the treating center. The foundation for the decision included individual maximal curative approaches where either a single remaining or non-regressive lesion at the time of RT (*n* = 9) or all MIBG-positive lesions at the time of RT (*n* = 8) were irradiated, a metastasis at a critical location (*n* = 4), and symptoms (*n* = 3). As a result of this decision process, out of 53 metastases present at the time of radiotherapy, 24 lesions were irradiated. Thereby, 1–3 lesions per patient received RT with a median dose of 36 Gy (range 20–45 Gy; Table 2).

**Table 1:** Patient and tumor characteristics

*Follow-up entire cohort, months, median (range)*	56.5 (16–210)	
*Gender*		
Male	13	72.2%
Female	5	27.8%
*Age at time of diagnosis*		
<18 months	3	16.6%
>18 months	15	83.4%
*NMYC*		
w/ amplification	3	16.7%
w/o amplification	14	77.8%
Unknown	1	5.5%
*Risk stratification according INRG*		
High risk	16	88.9%
Intermediate risk	2	11.1%
*INSS staging*		
Stage IV	18	100%
*No. of bony metastases at diagnosis, median (range)*	4.5 (1–11)	
*Bony metastases with RT*	3 (0–8)	
*Status of the primary tumor at the time of RT*		
Complete remission	13 (73%)	
Very good partial remission	2 (11%)	
Partial remission	2 (11%)	
Not applicable	1 (5%)	

*w/* with, *w/o* without, *RT* radiotherapy, *INRG* International Neuroblastoma Risk Group, *INSS* International Neuroblastoma Staging System

**Table 2:** Treatment characteristics

	No. of patients	
*HDCT*		
Yes	2	11.1%
No	16	88.9%
*Consolidation with anti-GD2 antibodies*		
Yes	12	66.7%
No	6	33.3%
*MIBG therapy*		
Yes	10^a^	55.6%
No	8	44.4%
*Radiotherapy of metastasis, median (range)*		
Total dose (Gy)	36 (20–45)	
No. of fractions	20 (12–30)	
Dose per fraction (Gy)	1.8 (1.5–2)	
No. of irradiated metastases per patient	1 (1–3)	
*Radiotherapy of the primary tumor*		
Yes	2	11.1%
No	16	88.9%

*HDCT* high-dose chemotherapy, *MIBG* 131-I-meta-iodobenzylguanidine

^a^Median time between MIBG therapy and radiotherapy 110 days (range 3–478)

### Treatment outcome

**Fig. 1:**
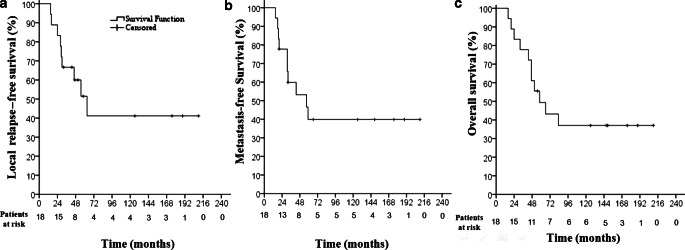
Kaplan–Meier analysis of local relapse-free survival (**a**; considering failure at irradiated metastatic sites), metastasis-free survival (**b**; considering only failure at non-irradiated metastatic sites), and overall survival (**c**) for the entire cohort

Median follow-up time was 56.5 months (range 16–210) for the entire cohort and 149 months (range 55–210) for survivors. Among the entire cohort, the LRFS rate at 2 and 5 years was 83.3 and 51.4%, respectively, and the MFS after 2 and 5 years was 77.8 and 39.9%, respectively (Fig. 1). The 2‑ and 5‑year OS rates were 72.2 and 49.4%, respectively. Thirteen of 18 patients relapsed at metastatic sites (Table 3). However, five patients were free of relapse or progression. All 13 patients with progression at metastatic sites progressed osteomedullary. In nine patients, progressions in the region of the irradiated site were observed, among them five with other progressive osteomedullary sites and four isolated at irradiated sites. Five patients progressed osteomedullary, but not at the irradiated sites. In four patients, combined with osteomedullary metastasis, soft tissue metastasis simultaneously occurred.

**Table 3:** Overview of the osteomedullary metastases, pattern of relapse, and survival status of the patients

Case	Osteomedullary lesions at time of RT (no.)	Irradiated osteomedullary lesions (no.)	Site of the irradiated metastasis	RT dose to metastatic sites	Local relapse (irradiated metastatic sites)	Metastatic relapse (non-irradiated site)	Failure of primary tumor site	Time to event or follow up (months)	Survival status
1	8	1	Skull	20.00 Gy	Yes	No	No	16	D
2	0	1	Skull	21.60 Gy	No	No	No	126 (no event)	AL
3	8	3	Vertebral body extremity	36.00 Gy	Yes	No	No	63	D
4	3	3	Extremity	45.00 Gy	Yes^b^	No	No	28	AL
5	2^a^	1	Vertebral body	39.60 Gy	No	No	No	210 (no event)	AL
6	3	1	Vertebral body	30.80 Gy	Yes	No	No	30	D
7	1	1	Rip	30.40 Gy	Yes	Yes	Yes	19	D
8	2	1	Skull	36.00 Gy	Yes	Yes	Yes	31	D
9	1	1	Skull	39.60 Gy	Yes	Yes	No	15	D
10	3	2	Extremity	36.00 Gy	Yes	Yes	No	29	AL
11	1	1	Skull	36.00 Gy	No	No	No	175 (no event)	AL
12	3	1	Extremity	40.00 Gy	No	No	No	189 (no event)	AL
13	3	1	Extremity	40.00 Gy	Yes	Yes	No	20	AL
14	4	2	Extremity	40.00 Gy	No	Yes	No	32	D
15	4	1	Skull	36.00 Gy	No	No	No	32 (no event)	D*
16	5	1	Skull	39.60 Gy	No	Yes	No	18	D
17	1	1	Extremity	36.00 Gy	No	Yes	No	57	D*
18	1	1	Rip	40.00 Gy	No	Yes	No	43	D

*RT* radiotherapy, *AL* alive, *D* deceased due to disease, D* deceased due to complications of the systemic therapy

^a^In addition to osteomedullary metastasis, the patient showed an MIBG-positive soft tissue lesion at the liver

^b^Re-irradiation was performed at recurrence with another 30 Gy leading to local metastatic control until last follow up (119 months after relapse)

At the time of analysis, 11 patients had deceased, nine due to tumor progression and two due to complications of the systemic therapy (*n* = 2). Dose of radiotherapy was not statistically evident to correlate with LRFS, MFS, or OS (*p* = 0.3). In one patient, re-irradiation was performed at recurrence (following first EBRT of 45 Gy at first diagnosis) with another 30 Gy leading to local metastatic control until last follow-up (119 months after relapse). Furthermore, MIBG therapy prior to irradiation did not show significantly better control rates (*p* = 0.4).

No secondary malignancy was reported.

### Toxicity

No acute or late high-grade toxicity attributable to RT was reported. However, two patients had deceased due to complications of the systemic therapy.

## Discussion

So far, evidence on RT for metastatic NB is still scarce and only few reports investigate the role of RT for bone metastases. Our results show that optimal treatment for children with metastatic NB remains unclear. As relapses often occur at previously involved sites of disease, evaluating the value of first-line RT to metastatic sites of disease is of great importance.

In this analysis of the prospective, multicenter German GPOH trials NB 97 and NB2004, we retrospectively evaluated children treated with EBRT to metastatic lesions of the bone with or without concomitant irradiation to the primary region.

With a median follow up of 149 months (range 55–220), we saw an LRFS rate at irradiated metastatic sites of 51.4% at 5 years with nine out of 18 patients developing local failure. An isolated local failure at an irradiated metastatic site was recorded in only four patients. Distant metastases at non-irradiated sites occurred in nine patients including five patients who developed both recurrence at irradiated metastatic sites and distant metastases at non-irradiated sites.

Two previous studies compared irradiated and non-irradiated metastatic sites and could not report a significant positive effect for RT to metastatic lesions. Polishchuck et al. analyzed the pattern of metastatic recurrence in 43 NB patients with a median age of 3.3 years. The majority of metastatic lesions were treated with a dose of 21.6 Gy in 12 fractions. However, the risk for recurrence was not statistically lower in irradiated metastatic sites compared to non-irradiated metastatic sites (*p* = 0.58) [10]. Similar results were found in a small study on stage 4 NB. Kandula et al. compared 13 patients receiving RT to one metastatic site per patient with 24 patients not receiving any RT for metastases concurrent with RT for the primary site. Irradiated metastases included bony as well as soft tissue metastases. Patients were treated with a median RT dose of 21.6 Gy (range 21–30.6 Gy) to the metastatic tumor visible after induction chemotherapy. The difference in 5‑year OS (73% with RT vs. 63% without RT) and relapse-free survival (46% with RT vs. 55% without RT) was not statistically significant. A local recurrence rate of 23% within the radiation field was reported [17].

In contrast to these reports, other non-comparative analyses published promising experiences. In a study performed at the Texas Children’s Hospital, 50 sites (*n* = 29 primary and *n* = 21 distant) were irradiated in 30 patients (maximum number of irradiated metastases per patient *n* = 3). Only 14 of the irradiated metastases were at bony sites. Patients received a dose of 24 Gy in 12 fractions to the residual metastatic tumor following induction therapy. An LC rate of 74% at 5 years for irradiated metastatic lesions was reported [9].

In a report of the Memorial Sloan Kettering Cancer Center, currently representing the largest study in RT for metastatic lesions in NB, 159 patients (1.2–17.9 years, mean age 4.0 years) were treated for 244 metastatic sites [8]. The majority of patients (85%) received hyperfractionated RT consisting of 21 Gy delivered in 1.5-Gy twice daily fractions to the tumor volume before induction chemotherapy. A 5-year LC rate of 81% for patients treated for metastatic sites was reported [8].

Although the sample size in our analysis is relatively small and hampers drawing a final conclusion, several differences might explain the comparatively low LRFS rate of 51.4% at 5 years in our cohort despite using an intensive RT regimen of a median 36 Gy to metastases compared to cohorts previously published. First, while in 17 of 18 patients of our study cohort the target volume was defined by the extension of the MIBG-active lesion at the time of RT, previous studies defined the target volume as the volume visible on cross-sectional studies either at initial staging or after induction chemotherapy. Due to these diverging approaches resulting in different target volumes, the comparison of data remains challenging [18–20]. There were no general recommendations for the irradiation of metastases within NB 97 and NB 2004 and the indication for RT to metastatic lesions was based on individual decisions, forming a risk for selection bias [21]. Additionally, in our cohort a significant number of lesions did not receive RT, which might be a reason for our low survival rates. Furthermore, the patients in our cohort had been treated in various institutes without any prospective quality control program and without quality assurance (QA). Therefore, there is a risk for non-homogeneous or even unsatisfactory RT approaches potentially compromising RT results [22].

The limited number of patients, impeding any statistical analysis, constitutes the main limitation of the present report. Furthermore, we critically acknowledge the retrospective analysis and the lack of a QA program. However, most of the above-mentioned reports are also observational, non-comparative cohort studies or included only a small number of patients [8–10, 17]. Nevertheless, the included patients were derived from two prospective trials. Due to the standardization in treatment and follow-up lasting for more than 10 years for the survivors, our cohort is remarkably homogeneous. However, within the framework of a multicenter study design, there is a potential risk that complications will remain underreported.

## Conclusion

Our study has shown that RT for children with high-risk NB metastases is feasible and highly tolerable, without any high-grade acute and late toxicity and no occurrence of secondary malignancies. In order to gain better evidence to clearly support the positive role of RT for residual osteomedullary metastatic lesions, data from larger cohorts are needed. In principle, randomized data would be desirable in order to identify any benefit in this critical patient group. Quality assurance (QA) programs for RT are essential in order to further improve treatment.

It remains unclear whether intensification of treatment by adding focal RT to osteomedullary metastatic sites is able to improve overall outcome in stage 4 NB. To date, an international consensus on RT of metastases of NB is still pending and might be explained by the paucity of convincing evidence.

However, the variety of treatment strategies and treatment results underline the importance of international consensus. In conclusion, in the absence of clear evidence, the indication for RT to metastatic lesions has to be reserved for multidisciplinary protocols or applied only after thoughtful individual multidisciplinary tumor board (MDT) decisions, in order to balance best chances and risks for these young patients.
